# Integrated Multiomics Analysis and Mendelian Randomization Identify SIRT1 as a Pivotal Aging‐Associated Gene in Meningioma

**DOI:** 10.1002/iub.70072

**Published:** 2025-11-10

**Authors:** Guangyu Du, Daikang Xu, Jingxian Sun, Shusheng Che, Junwei Ma, Xiaolei Lan, Jianpeng Wang, Zhiyong Yan

**Affiliations:** ^1^ Department of Neurosurgery The Affiliated Hospital of Qingdao University Qingdao Shandong China

**Keywords:** aging‐associated genes, immune infiltration, Mendelian randomization, meningioma, SIRT1

## Abstract

Meningiomas (MGMs) are the most prevalent benign intracranial tumors in adults, with incidence markedly increasing with age, underscoring the need to explore aging‐associated molecular mechanisms. In this study, we integrated transcriptomic datasets (GSE43290, GSE54934, GSE77259, and GSE183655) from the GEO database and aging‐related genes (ARGs) from the Human Aging Genomic Resources to identify key genes implicated in MGM. We screened differentially expressed ARGs (ARG‐DEGs) and conducted GO and KEGG pathway enrichment analyses, revealing significant involvement in cancer‐related processes, viral infection pathways, and the FoxO signaling pathway. Using LASSO, SVM, CytoHubba‐MCC, and MCODE algorithms, we identified two hub ARGs, SIRT1 and CEBPB. Immune infiltration analysis via ssGSEA indicated notable alterations in B cells, neutrophils, helper T cells, and regulatory T cells between MGM and healthy tissues, all closely associated with the hub genes. Furthermore, construction of a miRNA–TF–mRNA regulatory network highlighted the complex upstream regulation of these genes. Mendelian randomization analysis supported a potential causal relationship between SIRT1 and MGM development. Single‐cell RNA sequencing data further confirmed heterogeneous expression of SIRT1 across key cell populations within MGM, brain–tumor interface, and dura mater tissues. These findings were validated through qRT‐PCR and Western blot analyses, which demonstrated significant differences in SIRT1 expression at both the transcript and protein levels. Collectively, our study reveals that aging and immune dysregulation contribute to MGM pathogenesis and highlights SIRT1, in particular, as a potential diagnostic biomarker and therapeutic target, offering new insights into age‐related mechanisms underlying MGM.

## 1 Introduction

Meningiomas (MGMs) constitute one of the most prevalent primary central nervous system (CNS) tumors. Epidemiologic analyses indicate MGMs account for approximately 36.4% of all CNS neoplasms [1]. Over 90% of MGMs are benign [2], whereas approximately 1% demonstrate malignant characteristics. The recurrence rate of MGMs ranges between 10% and 20% [3]. Recent epidemiologic trends reveal progressive increases in both incidence and mortality rates [1], establishing MGMs as significant threats to human health among CNS tumors. Given the intrinsic pathological heterogeneity of these tumors, elucidating molecular pathogenesis and identifying robust biomarkers are paramount for enabling early detection, precise histological grading, prognostic recurrence evaluation, and personalized therapeutic strategies. This research further bears substantial socioeconomic implications for mitigating the escalating global healthcare burden associated with MGM management.

Cellular senescence—a self‐protective mechanism inherent in cellular evolutionary trajectories—increasingly demonstrates pivotal roles in pathophysiological processes including tissue injury, tumorigenesis, and aging [4]. Upregulation of senescence‐associated and senescence‐inducing genes occurs across diverse tumors. Such alterations drive tumor microenvironment modifications that may promote both carcinogenesis and malignant transformation of indolent tumors [5, 6, 7]. Investigations regarding brain tumor associations with human aging‐related genes continue to advance. Clinical studies reveal elderly individuals comprise the majority of glioma patients, exhibiting enhanced tumor invasiveness, treatment resistance, and diminished therapeutic efficacy [8, 9]. The mechanistic basis through which senescence influences MGM development and clinical prognosis remains incompletely characterized.

Existing evidence confirms MGM associations with human aging. Annual intracranial MGM incidence reaches 8.5 cases per 100,000 in elderly populations, contrasting with 2.5 cases per 100,000 in the general population [10], indicating substantially elevated occurrence among older individuals. Age‐related epigenetic alterations and somatic mutations further represent critical genetic features of MGMs [11]. Aging‐associated biological processes are intricately implicated in MGM initiation, progression, and prognosis.

Inflammaging exhibits close associations with neuro‐oncological pathologies. This phenomenon denotes sterile, chronic, low‐grade inflammation characterized by heightened cytokine production and reactive oxygen species generation [12]. Beyond reflecting chronological aging, it serves as both a biomarker of biological aging and an indicator of elevated mortality risk [13]. Substantial evidence demonstrates that CNS inflammation associated with aging promotes brain tumor development and metastasis [14, 15]. Cellular senescence—a biological aging marker with distinct systemic and clinical relevance [16]—represents a key factor in multiple tumor etiologies, including primary CNS malignancies [17]. Senescent cells secrete factors comprising the senescence‐associated secretory phenotype (SASP), which modulates tumor microenvironments via autocrine‐paracrine signaling. Significant senescent cell accumulation occurs in diverse CNS tumors including craniopharyngiomas, low‐grade gliomas, glioblastoma (GBM) multiforme, medulloblastomas, and diffuse midline gliomas. SASP components promote tumor progression by remodeling microenvironments, amplifying inflammatory responses, and facilitating tumor cell survival and metastasis [18]. Consequently, age‐related senescence of neural and stem cells accelerates tumor initiation and progression.

Aging constitutes a fundamental tumorigenic factor, with accumulating evidence implicating senescence‐associated genes in regulating both cellular senescence and tumor malignancy. Glioma‐focused clinical studies confirm significant associations between senescence‐related genes (e.g., LEP, TERT, PON1, SSTR3) and tumor progression, establishing their roles as therapeutic targets [19]. Basic research identifies NLRP3 inflammasomes as crucial molecular connectors between brain aging and glioma progression, with NLRP3 proposed as a predictive glioma biomarker [20]. NLRP3 inhibition suppresses senescence‐related gene expression, thereby attenuating glioma proliferation.

Collectively, these findings support utilizing epigenetic modifications to investigate physiological aging influences on brain tumorigenesis [7]. Genomic instability and epigenetic alterations constitute hallmarks of CNS tumor development and represent promising therapeutic targets. This study employs multidimensional bioinformatics methodologies to elucidate interrelationships among aging‐associated genes (ARGs), the immune microenvironment, and MGM pathogenesis.

## 2 Methods and Materials

### 2.1 Data Acquisition

This analysis integrated data from three GEO datasets. The primary transcriptomic dataset, GSE43290 [21], encompasses gene expression profiles (GEPs) from 47 MGM tissues and 4 normal meningeal samples, sequenced on the GPL96 platform. Dataset GSE54934 [22] contains transcriptomic data from 22 MGM tissues and 3 normal meningeal controls via the GPL6244 platform. Dataset GSE77259 [23] comprises 14 MGM tissues and 3 normal meningeal samples, similarly sequenced on GPL6244. Additionally, single‐cell RNA sequencing data (scRNA‐seq, GSE183655) were included, featuring six MGM samples, two brain‐tumor interface (BTI) specimens, and two normal dura mater samples sequenced on GPL24676.

### 2.2 Identification of ARGs

We retrieved human ARGs from the Human Aging Genomic Resources (HAGRs, https://genomics.senescence.info/) database [24]. This dataset includes genes from GenAge (307 genes) and CellAge bulid 3 (866 genes). After integration and removal of redundant genes, a total of 1061 ARGs were compiled for further analysis. The list of ARGs used in this study is provided in the Supporting Information.

### 2.3 Identification of Differentially Expressed Genes (DEGs) and ARG‐DEGs

DEGs were identified using the “limma” R package. To balance exploratory sensitivity with statistical rigor, an initial filtering was applied using thresholds of |log_2_FC| > 0.2 and a nominal *p*‐value < 0.05 to retain potentially biologically relevant genes. Subsequently, the Benjamini–Hochberg (BH) false discovery rate (FDR) correction was rigorously applied to control for multiple comparisons. Only genes with an FDR‐adjusted *p*‐value < 0.05 were considered statistically significant and retained for downstream functional and pathway analyses. The results were visualized using the “ggplot2” and “pheatmap” R packages. Subsequently, differentially expressed ARG‐DEGs were identified by extracting overlapping genes between ARGs and DEGs.

### 2.4 Functional Enrichment Analysis

Functional enrichment analysis was performed using the “ClusterProfiler” R package to conduct KEGG pathway and GO enrichment analyses for DEGs and ARG‐DEGs. The significance of enrichment was assessed via hypergeometric testing, and the resulting *p*‐values were subjected to FDR correction. Terms and pathways with an FDR‐adjusted *p*‐value < 0.05 were deemed significantly enriched, were subsequently visualized using the “ggplot2” and “Goplot” R packages.

### 2.5 Identification of Key ARG‐DEGs

In this study, three machine learning algorithms were employed to identify key ARG‐DEGs: least absolute shrinkage and selection operator (LASSO), support vector machine‐recursive feature elimination (SVM‐RFE), and random forest. All models were implemented in RStudio (version 4.2.1).

Feature selection via LASSO regression was implemented using the “glmnet” package in RStudio. A binomial regression model was applied. The optimal value of the regularization parameter *λ* was determined through 10‐fold cross‐validation, with the value yielding the minimum mean cross‐validated error (*λ*.min) selected for the final model. The analysis was repeated 100 times to ensure the stability of the selected features.

Features with nonzero coefficients in the final model were retained as robust predictors. The SVM‐RFE algorithm was executed using the “e1071” package. A linear kernel was employed for the SVM classifier. The recursive feature elimination process was conducted over five iterations of 10‐fold cross‐validation. In each iteration, features were ranked based on the absolute magnitude of the SVM weights, and the bottom 10% of features were sequentially eliminated. The final feature subset was determined as the set that consistently yielded the highest cross‐validated classification accuracy across all iterations.

Furthermore, the Random Forest algorithm was implemented using the “randomForest” package in R. An ensemble of 1000 decision trees (ntree = 1000) was grown for each model. The number of variables randomly sampled as candidates at each split (mtry) was tuned using the “caret” package; the default value (sqrt(total features)) was initially set and optimized via 10‐fold cross‐validation. The Gini impurity index was used for node splitting. The out‐of‐bag (OOB) error rate was calculated to assess model performance and feature importance. The final feature set was comprised of variables with a mean decrease in Gini importance above the median value across all features.

Protein–protein interaction (PPI) analysis was performed using the STRING database to explore the ARG‐DEGs and uncover potential gene relationships. To identify key nodes within the network, the CytoHubba‐MCC and MCODE plugins were utilized to filter out the critical ARG‐DEGs, which displayed high correlation within the PPI network. Subsequently, the results from the selected machine learning algorithms and the two CytoHubba plugins were cross‐validated to pinpoint the most crucial ARG‐DEGs.

After identifying the key ARG‐DEGs, to enhance the clinical applicability of GSE43290 dataset, a validation was performed using the GSE54934 and GSE77259 datasets.

### 2.6 Immune Microenvironment Deconvolution and Functional Analysis

To comprehensively characterize the heterogeneity of the tumor immune microenvironment (TIME) in MGM, we performed a computational deconvolution of bulk transcriptomic data using a MGM scRNA‐seq dataset as a reference. This approach allowed us to infer the relative abundances of distinct immune cell subsets within bulk tumor tissues.

The cellular composition analysis was conducted using the cell‐type identification by estimating relative subsets of RNA transcripts (CIBERSORT) algorithm, with the leukocyte gene signature matrix (LM22) defining 22 human immune cell phenotypes. The bulk RNA‐seq dataset GSE43290, comprising transcriptomic profiles from MGM and normal control tissues, served as the input for deconvolution. To enhance the biological relevance and accuracy of the deconvolution for MGM, we leveraged the publicly available scRNA‐seq dataset GSE183655. This dataset was processed and annotated to identify major immune cell populations, thereby providing a disease‐specific context for the interpretation of the LM22 signature.

The CIBERSORT analysis was executed with 1000 permutations for statistical robustness. To account for multiple testing across all 22 immune cell types, the *p*‐values derived from the deconvolution were adjusted using the BH method for controlling the FDR. Results were filtered to include only samples with an FDR‐adjusted *p*‐value < 0.05 for subsequent high‐confidence analyses. We then compared the estimated proportions of the 22 immune cell types between MGM samples and normal controls.

To further complement the cellular deconvolution and assess the overall functional state of the immune microenvironment, we performed single‐sample gene set enrichment analysis (ssGSEA) using the “GSVA” R package. This method quantified the enrichment scores of various immune‐related gene signatures, allowing for a comparison of immune‐related functional activity levels between the MGM and control groups. Statistical significance of the differences in ssGSEA scores was evaluated using the Wilcoxon rank‐sum test.

Furthermore, to functionally interrogate the immune microenvironment at a gene level, we investigated the expression profiles of two key ARG‐DEGs, across the inferred immune cell subsets, assessing the association between their expression levels and specific immune cell abundances.

### 2.7 Regulatory Network of Key ARG‐DEGs

We utilized miRTarBase to predict miRNAs targeting the key ARG‐DEGs, and employed the Enrichr database to identify transcription factors (TFs) associated with these key ARG‐DEGs, setting the threshold at *p* < 0.05. Subsequently, we constructed a miRNA‐TF‐mRNA regulatory network and visualized these networks using Cytoscape (version 3.9.0).

### 2.8 MR Analysis Clarified the Causal Relationship Between Key ARG‐DEGs and MGM

Mendelian randomization (MR) is a causal inference method based on genetic variation, which uses genetic variants as instrumental variables (IVs) to simulate the design of a randomized controlled trial, thereby inferring the relationship between exposure factors and outcomes. In this study, we performed two‐sample MR analysis using the “TwoSampleMR” R package to assess the causal relationship between key ARG‐DEGs and MGMs. We utilized expression quantitative trait loci (eQTLs) and protein quantitative trait loci (pQTLs) associated with MDD susceptibility. The eQTL summary‐level data were obtained from the IEU OpenGwas (https://gwas.mrcieu.ac.uk/) database [25], while the pQTL summary‐level data were sourced from the UKB ppp (https://www.ukbiobank.ac.uk/enable‐your‐research/register) database [26]. The summary‐level GWAS data for MGM prognosis were derived from the FinnGen (https://www.finngen.fi/fi) database [27]. The inverse variance weighted (IVW) method was used to calculate the effect estimates. The significance of the causal estimates was evaluated after applying FDR correction to the *p*‐values across all tested genetic instruments and outcomes. Associations with an FDR‐adjusted *p*‐value < 0.05 were considered evidence of a significant causal relationship.

### 2.9 Validation of Key ARG‐DEGs Expression in scRNA‐Seq Transcriptomes

We utilized the scRNA‐seq dataset GSE183655 to examine the expression of key ARG‐DEGs across different groups (MGM/BTI/dura). Data processing and analysis were performed using R package Seurat (v4.0) and visualized with ggplot2 (v3.4.0). The raw data were first subjected to quality control (QC), normalization, and integration, with cells filtered based on the following criteria: 500 < nFeature_RNA < 6000, 100 < nCount_RNA < 50,000, and percent.MT < 25%. The filtered data were then subjected to dimensionality reduction, with principal component analysis (PCA) performed and visualized using a DimPlot. Subsequently, t‐SNE dimensionality reduction and clustering were performed using Seurat::RunTSNE (perplexity = 30) and Seurat::FindClusters (resolution = 0.8), respectively. Cell type annotation was rigorously performed based on well‐established marker genes and functional classifications. We referred to the CellMarker database and MGM‐specific literature to define cell identities, resulting in 12 distinct subpopulations: Mesenchymal cytoskeletal, fibroblast‐like, macrophages, mesenchymal proliferating, macrophage low, CD8+ T cells, endothelial cells, oligodendrocytes, myeloid cells, pericytes, T cell subsets, and mesenchymal ECM. Cell type proportions and subpopulation distributions were quantified across predefined groups and visualized using several plot types via ggplot2. To validate key ARG‐DEGs, their expression levels were systematically compared across three groups within major cell types using Wilcoxon rank‐sum tests (adjusted *p* < 0.05), revealing group‐specific dysregulation patterns.

### 2.10 Collection of Human Tissue Samples

The dural tissue, BTI tissue, and MGM tissue samples used in this study were obtained intraoperatively from a single 58‐year‐old female patient undergoing surgery at the Department of neurosurgery, the affiliated hospital of Qingdao University. The normal dura mater sample was harvested from a site distal to the tumor. The BTI tissue was meticulously sampled from the transitional zone between the solid tumor mass and the adjacent normal‐appearing brain tissue. To ensure the pathological authenticity of the BTI sample and the exclusion of tumor cell infiltration, all collected tissues were immediately subjected to intraoperative frozen section analysis by a qualified pathologist. This study was approved by the Medical Ethics Committee of the affiliated hospital of Qingdao University. All samples were collected under sterile conditions and stored at −80°C until analysis.

### 2.11 RNA Extraction and Reverse Transcription

Total RNA was isolated using TRIzol Reagent (TIANGEN BIOTECH, China) following the manufacturer's instructions. RNA purity (1.8 ≤ A260/A280 ratio ≤ 2.1) and concentration were verified with a NanoDrop ND‐2000 spectrophotometer (Thermo Fisher Scientific, USA). First‐strand cDNA synthesis was performed with 1 μg RNA using the PrimeScript RT Reagent Kit (TaKaRa, RR047A) to eliminate genomic DNA contamination.

### 2.12 Quantitative Real‐Time PCR (qRT‐PCR)

SYBR Premix Ex Taq II (TaKaRa, RR820A) was used to quantify SIRT1 mRNA levels on an ABI 7500 real‐time PCR system (Applied Biosystems, USA). Primer sequences for SIRT1 and GAPDH (internal control) (sequences in Table S1) were designed using Primer Premier 5.0. Reaction conditions: 95°C for 30 s, followed by 40 cycles of 95°C (5 s) and 60°C (30 s). Relative expression was calculated using the 2^−ΔΔ*C*t^ method.

### 2.13 Western Blot

Total protein was extracted from tissues using RIPA lysis buffer (Beyotime, China) with 1 mM PMSF, and concentrations were determined via a BCA Protein Assay Kit (Thermo Fisher, USA). Proteins (30 μg/lane) were separated on 10% SDS‐PAGE gels and transferred to PVDF membranes (Millipore, USA). After blocking with 5% non‐fat milk, membranes were incubated overnight with primary antibodies: anti‐SIRT1 (1:1000, Zenbio, China, R25721) and anti‐GAPDH (1:10000, Abways, China, AB0036), followed by HRP‐conjugated secondary antibodies (1:10000, bioeasy, China, BE0101). Signals were detected using an ECL system (T4600, Tanon, China) and quantified via ImageJ.

## 3 Results

### 3.1 Identification of DEGs and KEGG/GO Pathway Analysis

**FIGURE 1:**
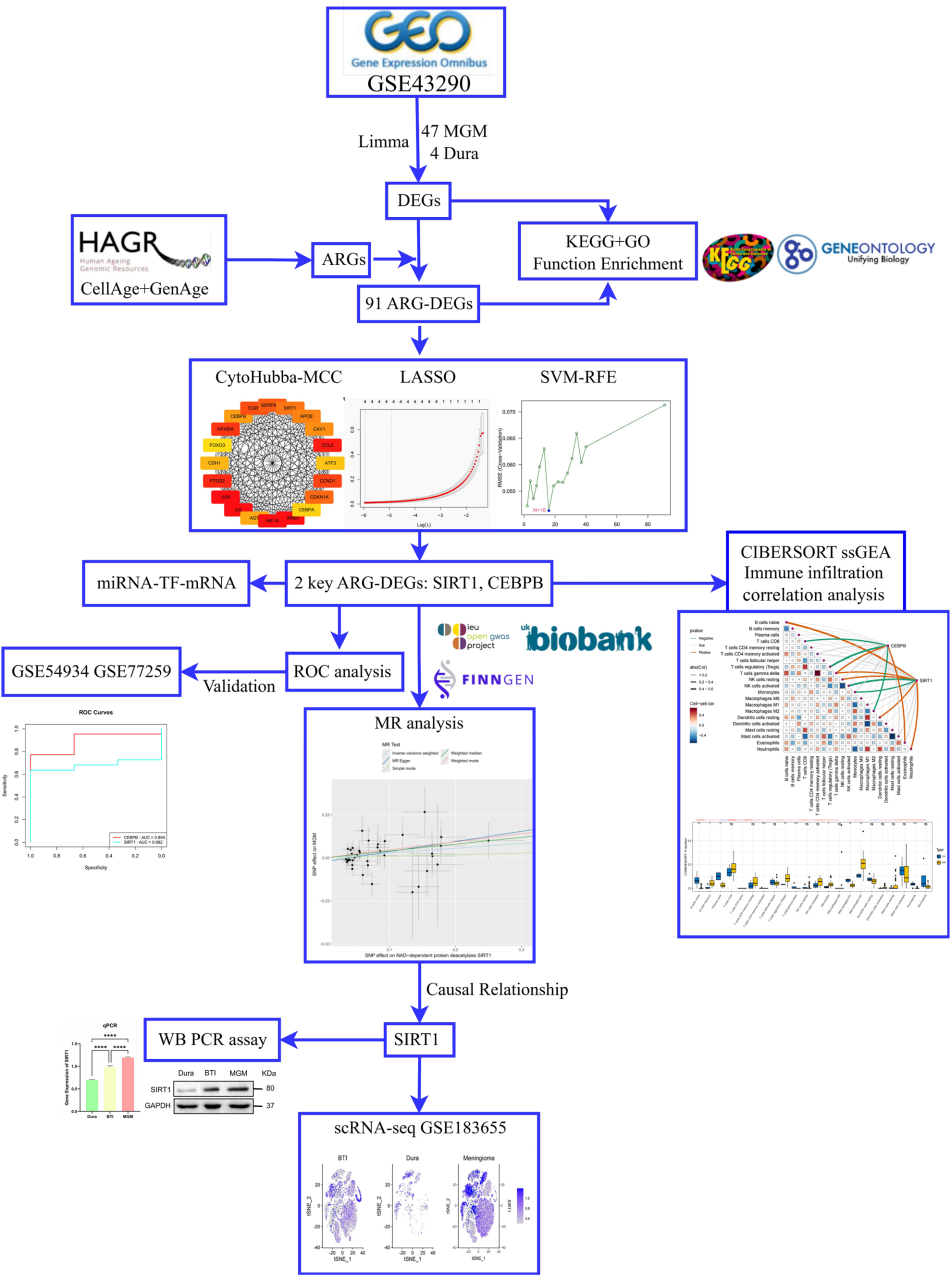
The flow chart of this study.

**FIGURE 2:**
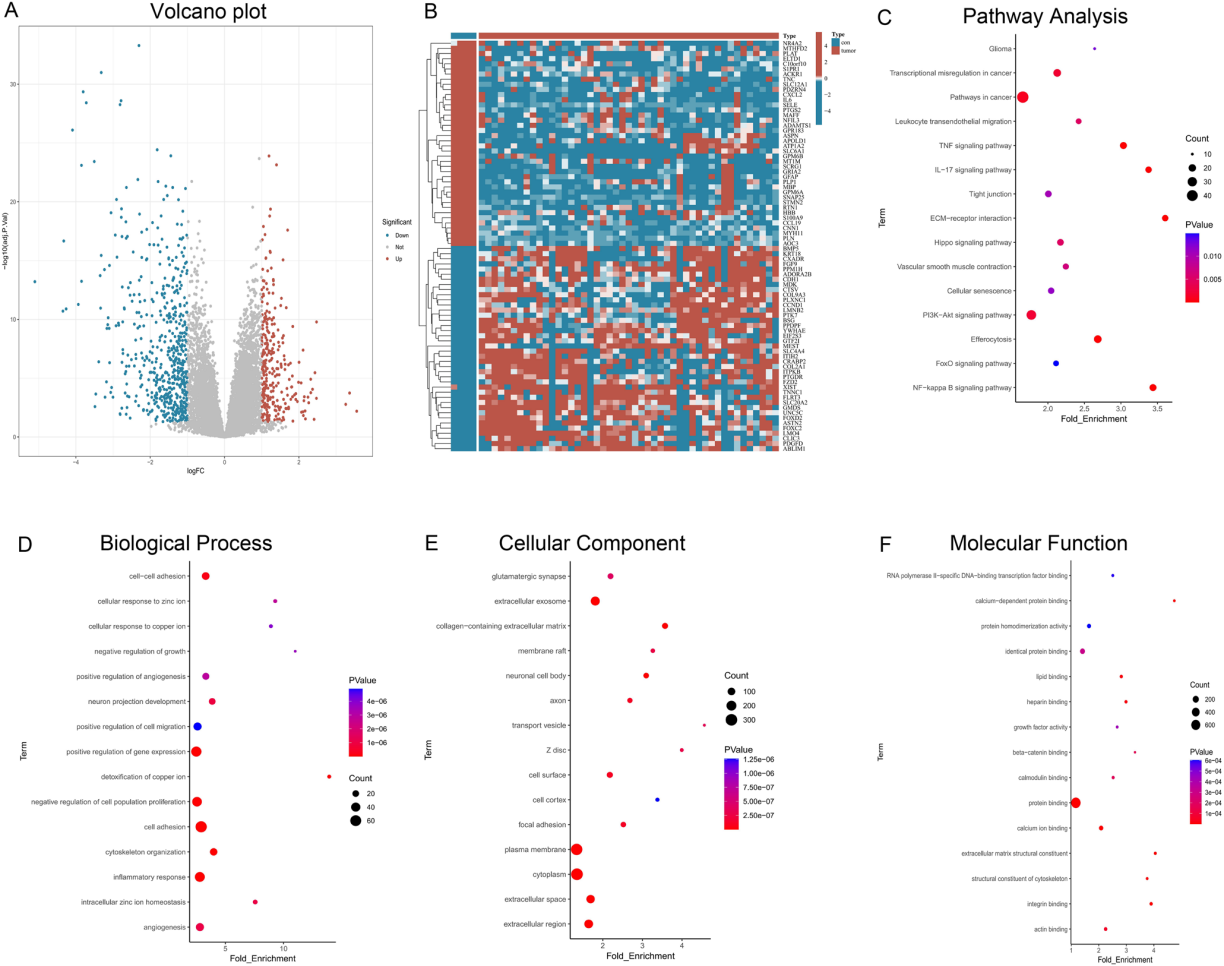
Identification and enrichment analysis of MGM‐DEGs. (A, B) Volcano plot and Clustered heatmap of DEGs in GSE43290; (C) KEGG pathway enrichment analysis of DEGs; GO enrichment analysis of DEGs: (D) BP (biological processes), (E) CC (cellular components), (F) MF (molecular function).

The research flow chart is shown in Figure 1. After preprocessing the original data of the dataset GSE43290, 858 differentially expressed genes were obtained (Figure 2A,B), including 291 upregulated genes and 567 downregulated genes.

Next, we conducted KEGG pathway and GO enrichment analysis of DEGs. We found that transcriptional misregulation in cancer, pathways in cancer, TNF signaling, PI3K − Akt signaling, efferocytosis, NF − kappa B signaling were the most highly enriched pathways (Figure 2C). Subsequently, we performed GO analysis to further characterize the functional roles of the DEGs. The results revealed that cell–cell adhesion, neuron projection development, positive regulation of gene expression, negative regulation of cell population proliferation, cell adhesion, inflammatory response, angiogenesis were among the signifcantly enriched BP (biological processes) (Figure 2D). In the cellular components (CCs) category, plasma membrane, cytoplasm, extracellular exosome were enriched item (Figure 2E). As for molecular function (MF), the signifcantly enriched terms were protein binding, identical protein binding (Figure 2F).

### 3.2 Identifcation of ARG‐DEGs and KEGG/GO Pathway Analysis

**FIGURE 3:**
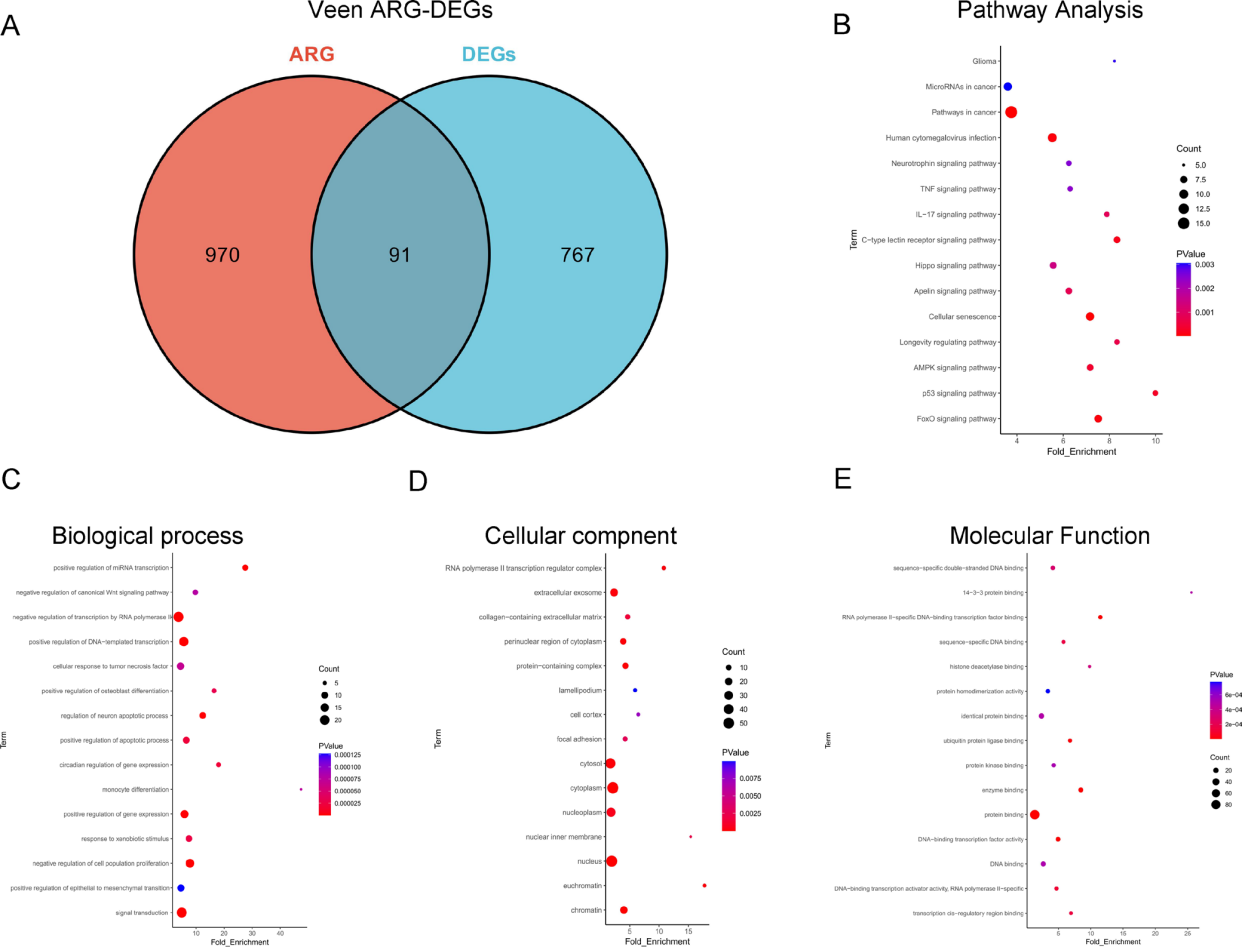
Identification and enrichment analysis of ARG‐DEGs. (A) Venn diagram of ARG‐DEGs in MGM and Control; (B) KEGG pathway enrichment analysis of ARG‐DEGs; (C–E) GO enrichment analysis of ARG‐DEGs.

By extracting the overlapping genes between ARGs and DEGs, we identified a total of 91 ARG‐DEGs (Figure 3A). We also performed KEGG pathway and GO enrichment analysis on ARG‐DEGs. The KEGG results revealed that pathways in cancer, human cytomegalovirus infection, cellular senescence, FoxO signaling pathway were the most signifcantly enriched pathways (Figure 3B). The BP results indicated that negative regulation of transcription by RNA polymerase II, positive regulation of DNA‐templated transcription, positive regulation of gene expression, signal transduction (Figure 3C). In terms of CC, the enriched item were cytosol, cytoplasm, nucleoplasm, nucleus (Figure 3D). Regarding MF, the most enriched term was protein binding (Figure 3E).

### 3.3 Identifcation of Key ARG‐DEGs Through Bioinformatics Analysis and Machine Learning

**FIGURE 4:**
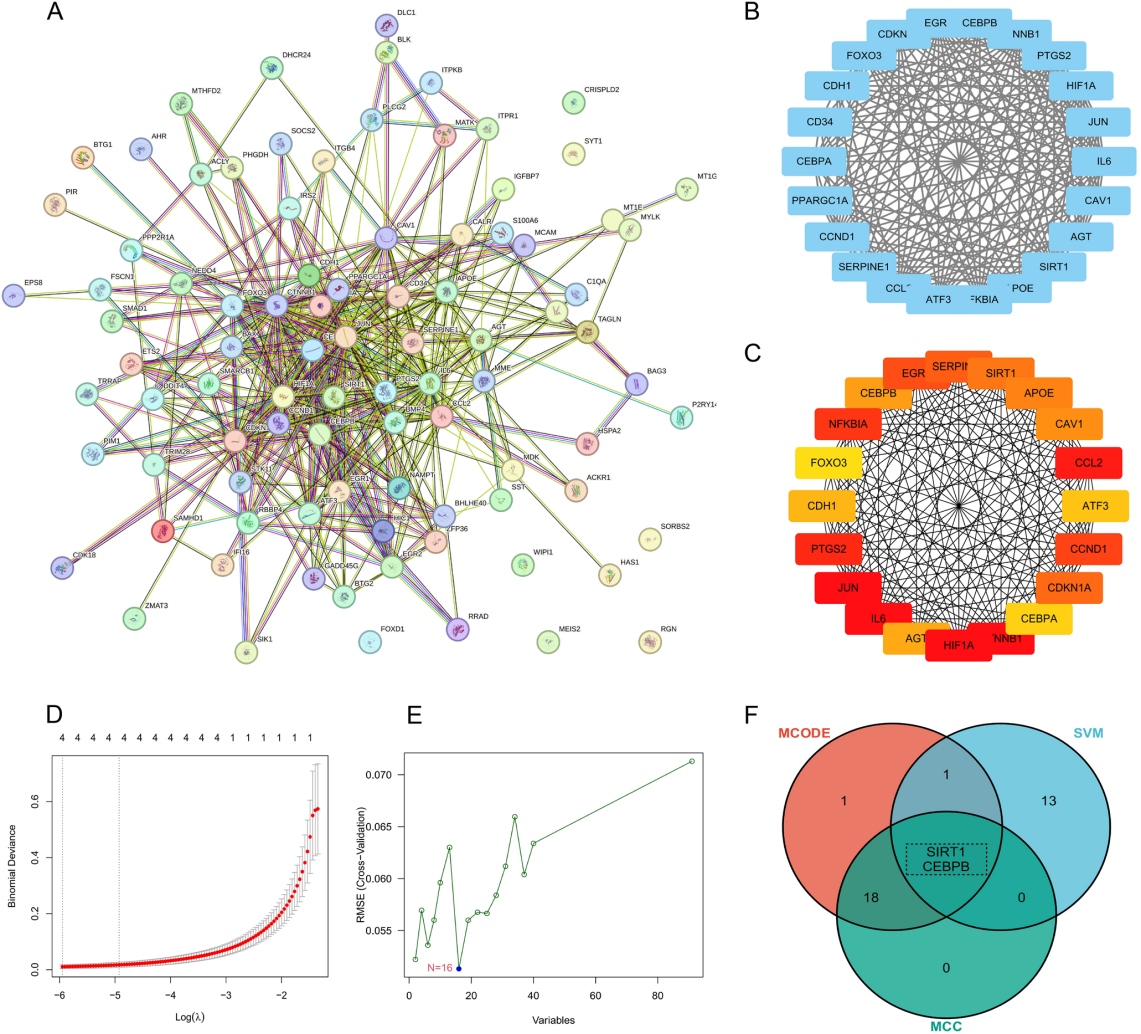
Identification of key ARG‐DEGs. (A) PPI network of ARG‐DEGs; (B) a key cluster with 22 genes was further chosen by MCODE; (C) Top 20 hub genes obtianed by CytoHubba‐MCC; (D) 20 hub ARG‐DRGs are screened by Lasso regression method; (E) 16 hub ARG‐DRGs are screened by SVM‐RFE method; (F) Venn diagram showed the genes intersection of SVM‐REF, MCC, MCODE.

In order to further identify key ARG‐DEGs, we initially performed a PPI network analysis on the 91 identified ARG‐DEGs and subsequently visualized the results (Figure 4A). Using the MCODE plugin, 22 key ARG‐DEGs were identified (Figure 4B), and then, the CytoHubba‐MCC plugin was employed to determine 20 pivotal ARG‐DEGs (Figure 4C). Subsequently, LASSO (Figure 4D), SVM‐RFE were applied to select key ARG‐DEGs (Figure 4E). The final two key ARG‐DEGs, namely SIRT1 and CEBPB, were identified through the integration of three methods (Figure 4F). Detailed results of these analyses are provided in Table S1.

**FIGURE 5:**
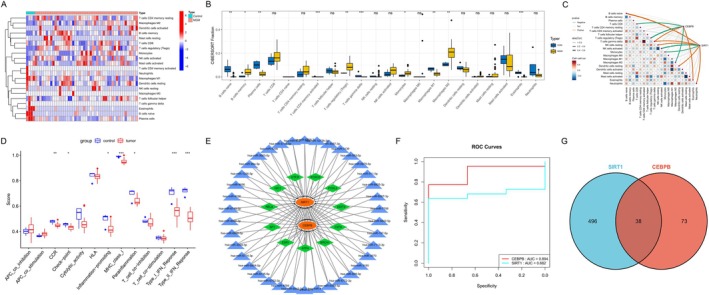
Immune characteristics of the MGM patients and healthy controls in the GSE43290 and GSE183655 (A, B) CIBERSORT analysis of differential immune cell infiltration between MGM samples and normal samples; (C) the correlation between key ARG‐DEGs and immune cells. (D) ssGSEA analysis of immune functions. (E, G) miRNA‐TF‐mRNA interactions regulating of two key ARG‐DEGs. (F) ROC curve analysis of key ARG‐DEGs in the GSE77259.

To evaluate the diagnostic accuracy of MGM using these two genes, ROC analysis was performed on the validation set GSE54934, revealing an AUC value of 0.682 for SIRT1 and 0.894 for CEBPB (Figure 5F). A similar ROC analysis was then conducted on the GSE77259 dataset for validation.

### 3.4 Immune Microenvironment of MGM Patients

We evaluated the differential expression of 22 immune cell types and 13 immune functions in MGM versus control samples using CIBERSORT and ssGSEA analyses. The results revealed significant dysregulation in 10 immune cell types in the MGM group, including: B cells naive, B cells memory, Plasma cells, T cells CD4^+^ memory activated, T cells regulatory (Tregs), T cells gamma delta, Monocytes, Macrophages M1, Macrophages M2, and Eosinophils (Figure 5A,B). Moreover, seven immune functions in MGM, namely CCR, Check‐point, Inflammation‐promoting, MHC class I, Parainflammation, and Type I/II interferon (IFN) Response, exhibited dysregulation compared to the control samples (Figure 5D). Both SIRT1 and CEBPB were associated with multiple immune cell types and functions. Specifically, SIRT1 showed a positive correlation with T cells—particularly, resting memory CD4^+^ T cells and Tregs—while exhibiting a negative correlation with myeloid cells. Conversely, high expression of CEBPB promoted the proliferation and activation of myeloid immune cells (Figure 5C).

### 3.5 The miRNA‐TF‐mRNA Regulatory Network Construction

To further investigate the regulatory mechanisms, we intersected the miRNAs associated with the genes SIRT1 and CEBPB, resulting in 38 key miRNAs (Figure 5G). Subsequently, we conducted predictive analysis on these 38 critical miRNAs and 11 TFs related to the two key ARG‐DEGs, forming a regulatory interaction network involving 49 miRNAs, TFs, and mRNAs. This complex regulatory network was then visualized for better representation (Figure 5E).

### 3.6 The MR Identified the Relationship Between SIRT1 and MGM

We further performed MR analyses, selecting IVs according to the following criteria: (1) we used a threshold of *p* < 5 × 10^−8^ to screen SNPs; (2) linkage disequilibrium (LD) was clumped using an *r* ^2^ < 0.001 and a window size of 10,000 kb; (3) SNPs with an *F*‐statistic < 10 were excluded to mitigate weak instrument bias, retaining only those with *F* ≥ 10; (4) palindromic SNPs with ambiguous strand orientation were removed; (5) SNPs associated with potential confounders were identified and excluded using PhenoScanner (www.phenoscanner.medschl.cam.ac.uk).

**FIGURE 6:**
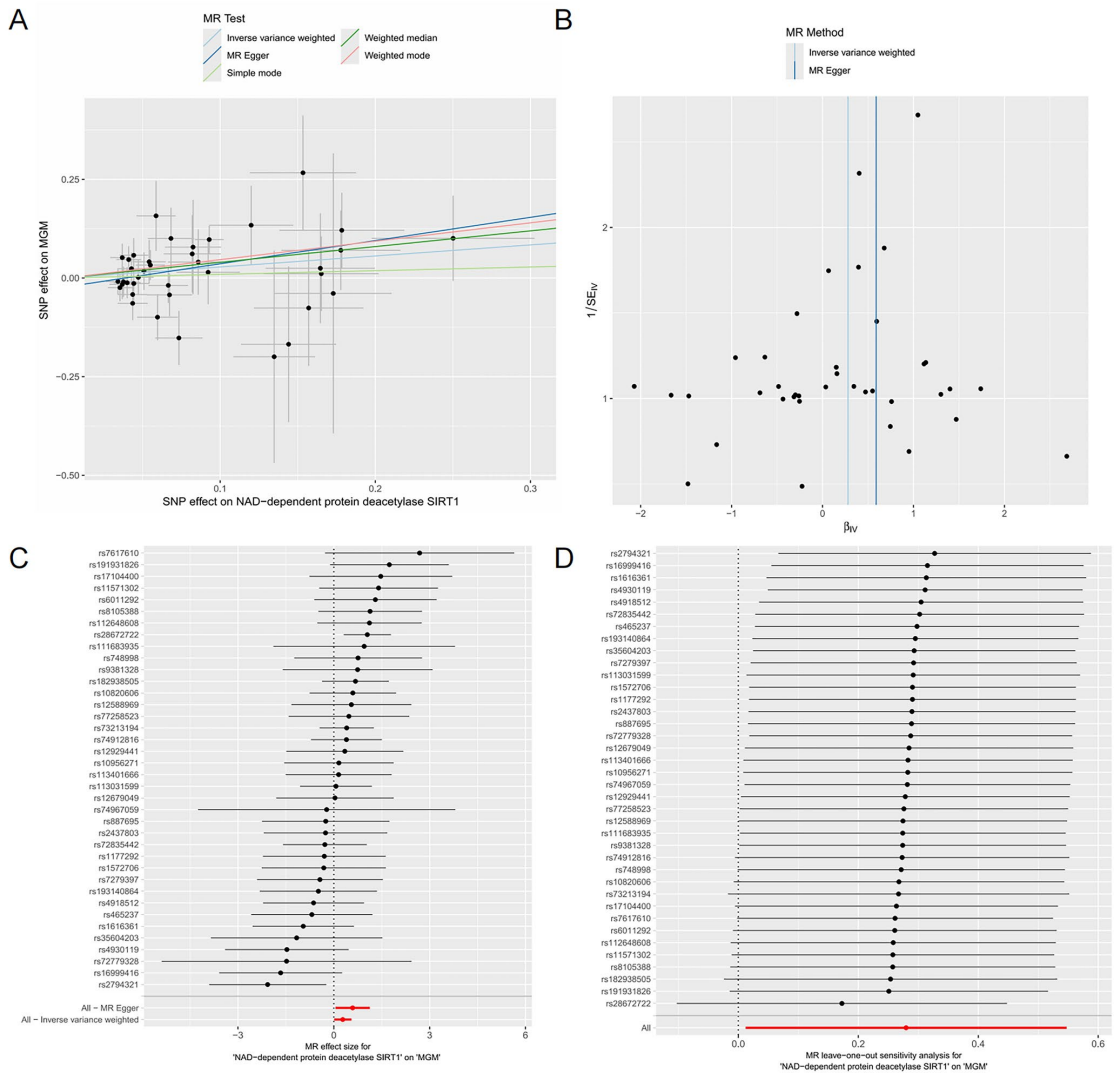
Results of Mendelian randomization analysis. (A) Results of MR Analysis of SIRT1 and MGM. The *x* axis represents the effect of the single nucleotide poly morphism (SNP) on exposure, and the *y* axis represents the effect of the SNP on the outcome. A slope > 0 indicates that SIRT1 is a harmful factor for MGM; (B) funnel plots show no heterogeneity of SNPS; (C) in the forest map of SNP, the comprehensive results (red line) can be seen that the higher the SIRT1, the higher the risk of MGM; (D) leave‐one‐out analysis of the causal association of SIRT1.

We observed an association between SIRT1 and the risk of MGMs in the eQTL data (IVW, OR = 0.86, 95% CI = 0.75–0.99, *p* = 0.031). Due to the limited number of SIRT1 SNPs identified in the pQTL data after filtering, MR Egger analysis could not be conducted. Consequently, we relaxed the selection threshold for IVs (*p* < 1 × 10^−5^), and further analysis revealed that SIRT1 proteomics data are also associated with the risk of MGMs (IVW, OR = 1.32, 95% CI = 1.01–1.73, *p* = 0.040). No causal relationship between CEBPB and MGMs was found in both eQTL and pQTL data (*p* > 0.05, Table S1). The Cochran's *Q* test indicated no evidence of heterogeneity in the MR results (*Q*_*p* > 0.05). Both MR‐Egger regression (*p* = 0.90) and MR‐PRESSO analysis (*p* = 0.097) indicated no significant pleiotropy at the overall level. These findings are illustrated through scatter plots (Figure 6). Additionally, leave‐one‐out analysis demonstrated that the overall estimates were not influenced by any single SNP.

### 3.7 The Expression of SIRT1 in scRNA‐Seq Transcriptomes

**FIGURE 7:**
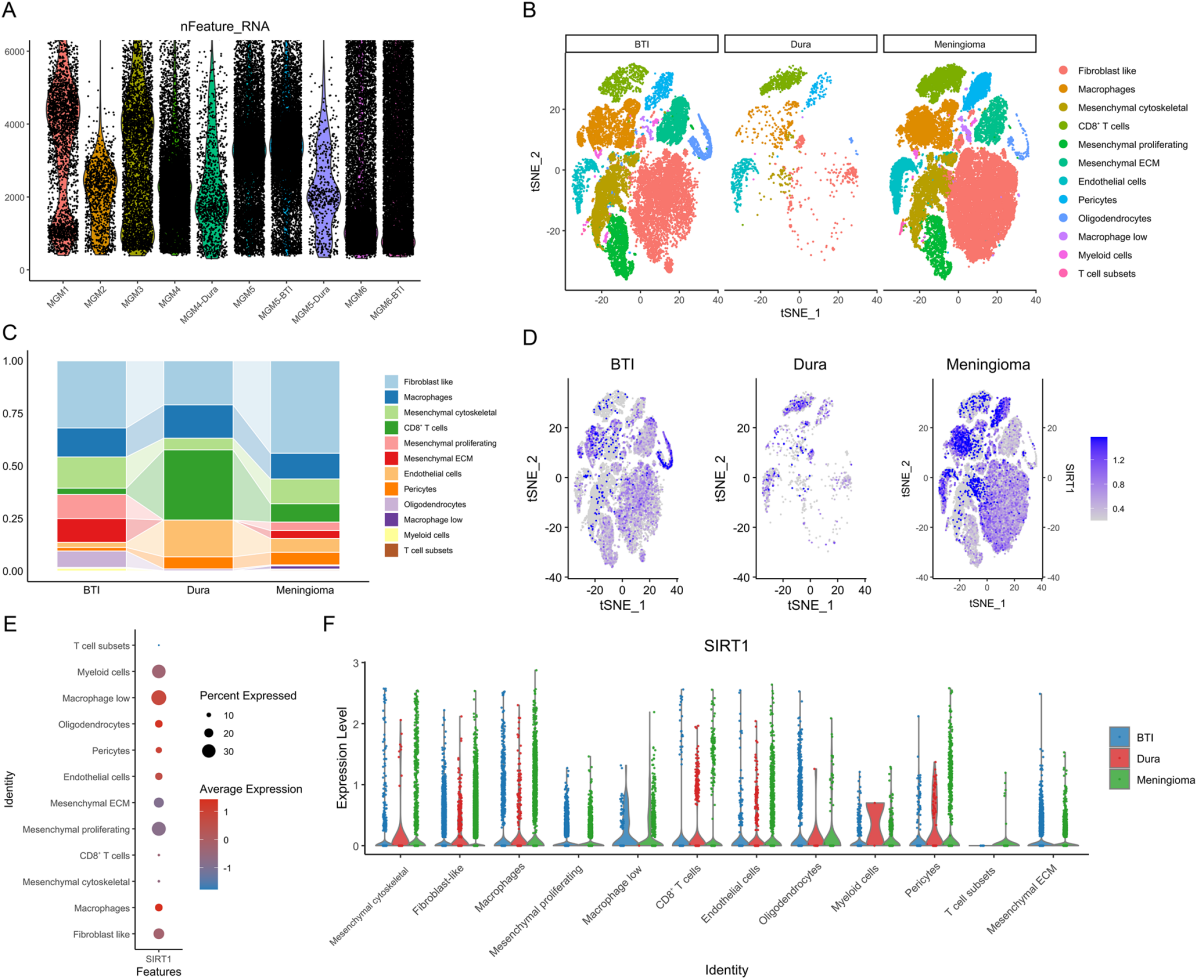
SIRT1 expression profile across BTI, dura, and meningioma (MGM) tissues in the GSE183655 scRNA‐seq dataset. (A) Violin plots showing the distribution of detected genes across samples from BTI, dura, and MGM tissues; (B) t‐SNE visualization of single‐cell transcriptomes colored by cell types (mesenchymal cytoskeletal, fibroblast‐like, macrophages, mesenchymal proliferating, macrophage low, CD8+ T cells, endothelial cells, oligodendrocytes, myeloid cells, pericytes, T cell subsets, and mesenchymal ECM) across BTI, dura, and MGM tissues; (C) stacked bar plots showing the relative proportions of different cell types within BTI, dura, and MGM groups, highlighting tissue‐specific cellular compositions; (D) feature plots displaying the expression of SIRT1 in t‐SNE space for each group; (E) dot plot summarizing SIRT1 expression across major cell types. Dot size indicates the percentage of cells expressing SIRT1, while color intensity represents the average expression level in each cell type; (F) violin plots showing the expression levels of SIRT1 in each annotated cell type across the three tissue groups (BTI in blue, dura in red, MGM in green).

To further explore the expression profile of the SIRT1 gene in MGM tissues and assess its specificity relative to normal meningeal tissue, we conducted a detailed analysis of SIRT1 expression using scRNA‐seq spatial transcriptomics data. For dataset GSE183655, the initial cell count prior to QC filtering was 80,225; following rigorous QC filtering, 58,417 high‐quality cells were retained for subsequent analysis (Figure 7A). The high‐dimensional data were subjected to PCA for dimensionality reduction, retaining top principal components that accounted for > 90% of cumulative variance. Following dimensionality reduction and graph‐based clustering, cell populations were annotated into 12 distinct cell types based on functional categories. Subsequent Tt‐SNE projection clearly segregated 12 functionally annotated cell types. In BTI samples, fibroblast‐like cells (red) formed prominent dense clusters, accompanied by substantial presence of macrophages (orange) and mesenchymal cytoskeletal cells (yellow). Dura controls exhibited relatively dispersed cell distribution with higher abundance of CD8^+^ T cells (green) and endothelial cells (light blue). MGM samples were dominated by fibroblast‐like cells and mesenchymal ECM cells (green), indicating strong stromal activation. Immune populations including T cell subsets (pink) and myeloid cells (light blue) were sparsely distributed across all groups (Figure 7B). Quantification of cell type proportions revealed significant differences across groups. Fibroblast‐like cells constituted the largest proportion in MGM (approximately 40%), followed by BTI (around 30%), and were lowest in dura (about 20%). Macrophages were enriched to a similar extent across the three tissue types (10%–20%), yet still displayed a trend of dura > BTI > MGM. Mesenchymal cytoskeletal cells were most prevalent in BTI (approximately 20%) and relatively reduced in the dura group (5%). CD8^+^ T cells and endothelial cells were significantly abundant in dura (collectively accounting for about 30%), but markedly decreased in both BTI and MGM. Myeloid cells and T cell subsets consistently demonstrated low proportions across all cohorts (Figure 7C).

The t‐SNE clustering reveals single‐cell heterogeneity. SIRT1 expression exhibited heterogeneous and context‐dependent distribution. In dura, expression was diffuse and covered multiple cell types at low intensity. BTI samples demonstrated moderate SIRT1 expression with emerging local clustering, particularly in regions rich in Macrophages and mesenchymal cells. MGM presented highly focused SIRT1‐high subclusters that topographically overlapped with fibroblast‐like and mesenchymal ECM regions, suggesting site‐specific upregulation within tumor‐associated stromal niches (Figure 7D). Among the 12 major CCs, macrophages and myeloid cells exhibited the highest proportion of SIRT1‐expressing cells and the strongest expression levels. Fibroblast‐like, mesenchymal cytoskeletal, and mesenchymal ECM cells showed an intermediate prevalence of SIRT1 expression, but their average expression levels ranged from moderate to low. Although a substantial subset of these stromal cells expressed SIRT1, the expression intensity within these populations was not particularly high. Endothelial cells and pericytes demonstrated relatively limited and weak SIRT1 expression. In contrast, T cell subsets and CD8^+^ T cells displayed the lowest expression levels, while oligodendrocytes showed very low prevalence and minimal expression, indicating that SIRT1 is rarely expressed and present at negligible levels in these lymphoid and neural cell populations (Figure 7E). Finally, we compared the expression levels of these cells across three distinct tissue types. The expression of SIRT1 demonstrates a clear cell type‐dependent pattern. Macrophages and myeloid cells consistently exhibit the highest expression levels across all three sample groups, indicating that SIRT1 plays an important role in these myeloid immune cells. In contrast, T cell subsets, CD8^+^ T cells, and oligodendrocytes consistently show the lowest or nearly absent SIRT1 expression, suggesting minimal involvement in lymphoid and neural lineages. In fibroblast‐like, mesenchymal cytoskeletal, and mesenchymal ECM cells, the MGM group (blue) frequently displays significantly elevated SIRT1 expression compared to the BTI (red) and dura (green) groups. Although expression is generally high across all groups, macrophages and myeloid cells in the MGM group also show a subtle yet consistent increase compared to the other groups. Endothelial cells and pericytes exhibit low to moderate expression, with the MGM group again demonstrating a mild elevation, potentially indicating altered SIRT1 activity within the tumor vascular system. For many cell types, the expression level appears highest in MGM samples (blue), followed by the BTI group (red), while the dura control samples (green) generally show the lowest SIRT1 expression levels. This overall pattern further validates the systematic upregulation of SIRT1 in the context of MGM pathology compared to non‐tumor control (Figure 7F).

### 3.8 Verification of SIRT1 High Expression in MGM by Western Blot and qRT‐PCR

**FIGURE 8:**
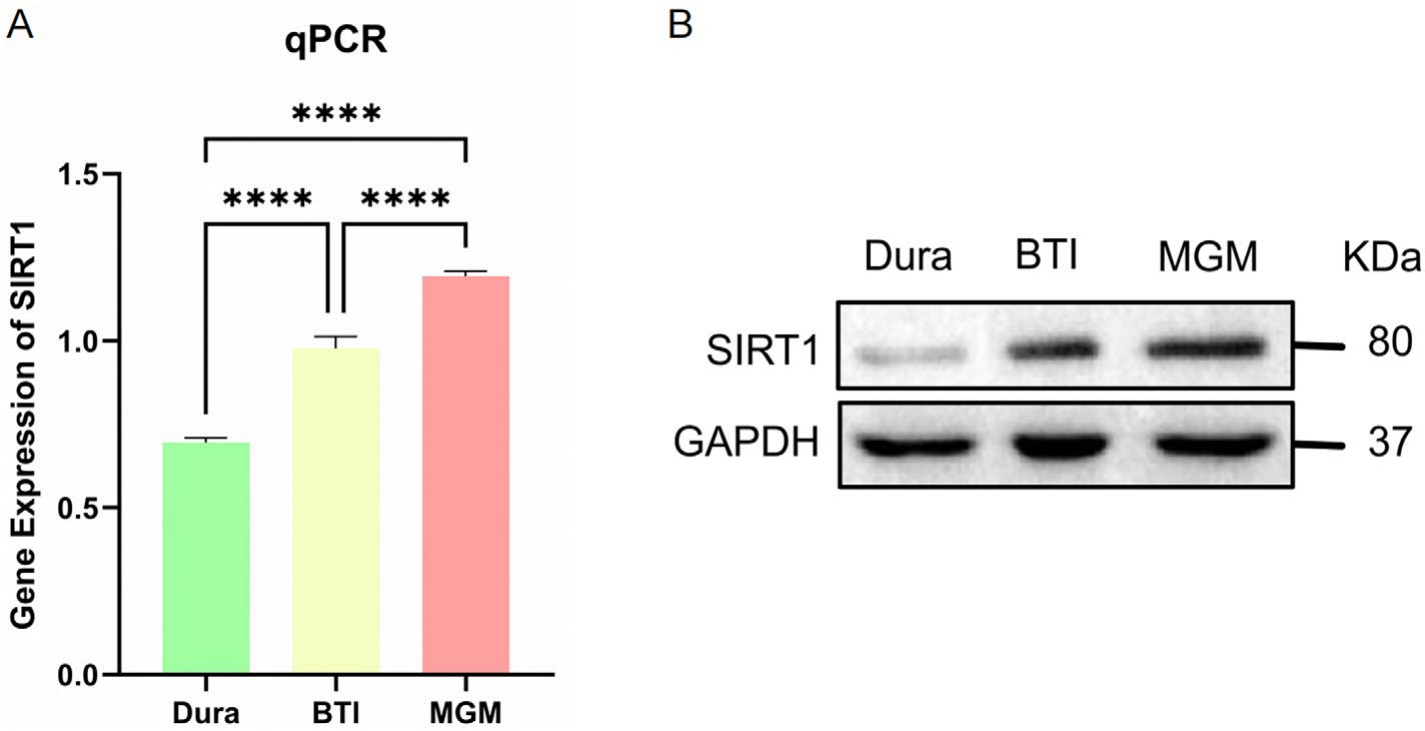
Validation of SIRT1 expression in dura, BTI, and Meningioma tissues by qRT‐PCR and Western blot. (A) Quantitative real‐time PCR (qRT‐PCR) analysis of SIRT1 mRNA expression in dura, BTI, and MGM tissues. SIRT1 expression was significantly upregulated in BTI and Meningioma compared to dura. MGM tissues showed the highest expression level. Data are presented as mean ± SEM. ****p < 0.0001; (B) Western blot analysis of SIRT1 protein expression in dura, BTI, and MGM tissues. Consistent with the qRT‐PCR results, SIRT1 protein levels were elevated in BTI and MGM samples compared to dura. GAPDH was used as a loading control.

Following the processing of the obtained human tissue samples, qRT‐PCR analysis revealed a progressive increase in the transcriptional expression levels of the SIRT1 gene from the dura mater through BTI to MGM tissues. In parallel, Western blotting analysis confirmed a corresponding gradual elevation in SIRT1 protein expression across the same tissue gradient (Figure 8).

## 4 Discussion

MGMs represent the most prevalent primary intracranial tumors in adults, yet research on their molecular biology and pathogenesis remains less extensive compared to other CNS tumors [28]. According to the Central Brain Tumor Registry of the United States (CBTRUS), nonmalignant MGMs exhibit the highest incidence among CNS tumors in the United States at 9.73 cases per 100,000 individuals. This incidence escalates beyond age 65 and further increases after age 85. Age‐adjusted rates of non‐malignant MGMs persistently rise across all genders, races, and ethnicities. In patients aged ≥ 20 years, MGMs constitute the most common intradural spinal tumors (39.9%), despite spinal variants comprising merely 4.2% of all MGMs [29].

Primary CNS tumors—exemplified by MGMs—demonstrate the highest incidence and mortality in elderly populations [10, 30]. Given the strong age‐dependency of primary nervous system tumor occurrence, decelerating aging correlates with reduced tumor incidence. Elevated incidence and mortality not only threaten elderly health but also impose substantial societal burdens. Although considerable clinical and epidemiological evidence supports the MGMs‐aging association, underlying mechanisms remain poorly characterized. Consequently, investigating shared biological characteristics between aging and primary nervous system tumors holds significant implications for disease prevention and treatment.

Aging and primary nervous system tumors exhibit converging pathobiological features, encompassing chronic inflammation, cellular senescence, stem cell depletion, genomic instability, epigenetic alterations, metabolic dysregulation, telomere dysfunction, immune impairment, aberrant intercellular communication, proteostasis disruption, autophagy suppression, and gut microbiota dysbiosis.

In this investigation, bioinformatics methodologies identified ARGs differentially expressed in MGM patients versus healthy controls (ARG‐DEGs). Two pivotal ARG‐DEGs—SIRT1 and CEBPB—were discerned, with subsequent analyses revealing their regulatory relationship. However, subsequent MR analysis to assess potential causal relationships with MGM development revealed robust genetic evidence supporting a causal role for SIRT1, whereas no significant causal association was observed for CEBPB. Given the priority for targets with genetic support, our subsequent experimental validation and mechanistic discussions focused exclusively on SIRT1. The lack of genetically predicted causal effects for CEBPB suggests that its differential expression may be a consequence of the disease or confounded by other factors, rather than a driving factor, thereby reducing its priority as a therapeutic target for MGM in this study. Single‐cell transcriptomic data further elucidated differential SIRT1 expression across MGM, BTI, and normal dura tissues.

The sirtuin (SIRTs) family comprises highly conserved NAD^+^‐dependent class III histone deacetylases (HDACs, SIRT1‐SIRT7) [31]. SIRT1 and SIRT2 exhibit high cerebral expression, with SIRT1 predominantly nuclear‐localized and demonstrating potent deacetylase activity [32]. SIRT1 modulates p53‐mediated transcription through direct p53 deacetylation or regulation of CBP/p300 acetyltransferases [33, 34]. Additional transcriptional targets include forkhead box (FOXO) family members, which govern critical cellular processes: proliferation, differentiation, cell‐cycle arrest, and survival [35, 36]. SIRT1 further regulates inflammatory factor transcription by suppressing NF‐κB signaling, thereby enhancing oxidative metabolism and inflammation resolution [37, 38]. Interactions with autophagy proteins (Atg5/7/8) coordinate autophagy‐lysosomal pathways [39, 40]. Owing to broad substrate specificity, SIRT1 critically modulates inflammation, oxidative stress, and apoptosis [41]. Initially studied in age‐related diseases, neurodegeneration [42, 43], inflammatory disorders [44], myopathies [45], and diverse cancers [46, 47, 48], SIRT1 is established as a key regulatory factor and therapeutic target in GBM multiforme pathogenesis.

SIRT1 transcription is governed by multiple TFs and cofactors. Our analyses predicted key SIRT1‐targeting miRNAs and associated TFs, including extensively studied repressor hypermethylated in cancer 1 (HIC1) and activator FOXO3a. SIRT1 expression dynamically adapts through negative feedback loops across cellular contexts [49]. HIC1 hypermethylation reduces its tumor expression, significantly upregulating SIRT1 [50]. HIC1 forms a repressive complex with SIRT1 and C‐terminal binding protein 1 (CTBP1), binding SIRT1 promoter enhancers to suppress transcription [51]. Concurrently, SIRT1 and HDAC4 mediate HIC1 deacetylation at lysine 314, promoting SUMOylation and inhibiting HIC1 complex activity [52]. FOXO3a enhances SIRT1 transcription via binding sites shared with p53. During pheochromocytoma treatment, acute nutrient stress triggers FOXO3a nuclear translocation and p53 interaction, displacing repressive complexes from the SIRT1 promoter [53]. Reciprocally, SIRT1‐deacetylated FOXO3a participates in cell‐cycle control, energy metabolism, and oxidative stress regulation [54, 55].

Based on the CIBERSORT immune infiltration analysis of MGMs, our study revealed that MGM tissues exhibit a distinct immune microenvironment profile. Compared to normal dura mater, MGMs showed significant downregulation of CD4^+^ resting memory T cells and Tregs, suggesting impaired Type I/II IFN response, which may undermine antitumor immune initiation and antigen memory function. Concurrently, a reduction in activated mast cells reflects suppressed TNF‐α‐mediated pro‐inflammatory signaling, further exacerbating defects in local immune responses. However, an increase in cytotoxic immune cells—such as activated CD8^+^ T cells, M1 macrophages, and gamma‐delta T cells—was also observed, indicating that the host immune system attempts to mount an antitumor response. This paradoxical state, characterized by coexisting infiltration of effector cells and inhibition of key immune‐activating pathways, highlights the dual features of immunoediting and immune escape during MGM progression. It also provides a rational basis for potential combination immunotherapy strategies, such as targeting inhibitory pathways while enhancing effector cell functions.

SIRT1 critically modulates inflammation, with its expression influenced by immune status. Substantial evidence indicates SIRT1 downregulation during acute inflammatory responses in vivo and in vitro. Upstream molecules exert anti‐inflammatory effects via SIRT1 upregulation [51, 56, 57, 58, 59, 60]. For instance, IFN‐γ disrupts skeletal muscle energy metabolism during chronic inflammation by inducing HIC1 and Class II transactivator (CIITA) [61]. TLR4‐induced endotoxin tolerance elevates SIRT1, inhibiting TNF transcription in THP1 cells [62]. In sepsis, SIRT1 accumulates at IL‐1β/TNF‐α promoters with NAD^+^‐enhanced H3K16 deacetylation [63]. SIRT1 also suppresses IL‐6/TNF‐α transcription through reduced H3K9 acetylation [63] and diminishes H3K16 acetylation at TNF‐α promoters during septic inflammation [64]. Collectively, these observations implicate SIRT1 in MGM immune‐inflammatory regulation.

SIRT1's multifunctionality—including immune‐inflammatory modulation—enables regulation of diverse tumorigenic processes. Evidence positions SIRT1 at multiple carcinogenesis stages: genomic instability, initiation, proliferation, metabolism, and therapeutic response [65]. Its impact exhibits context‐dependent duality: SIRT1 suppresses tumor initiation by promoting DNA repair, genomic stability, and anti‐inflammation during precancerous phases; conversely, it enhances proliferation, survival, and chemoresistance via antiapoptotic effects, metabolic reprogramming, and immunosuppression during progression/metastasis.

Among malignancies, hepatocellular carcinoma (HCC) represents the most comprehensively studied context for SIRT1. SIRT1 expression is significantly elevated in HCC versus adjacent tissue. As a longevity‐associated gene, it demonstrates microenvironment‐dependent functional duality. Elevated SIRT1 in liver cancer stem cells (CSCs) correlates with poor prognosis; its inhibition induces CSC senescence via p53‐p21/p16 pathways, reducing tumorigenicity and enhancing chemosensitivity [66]. SIRT1 regulates CSC self‐renewal genes (e.g., SOX2) through epigenetic modifications [66] and modulates autophagy to impact HCC progression and drug resistance [67]. Contrastingly, SIRT1 loss increases DNA damage to accelerate HCC [68].

In GBM, SIRT1 displays analogous regulatory complexity: under physiological stress, it promotes neuronal survival through DNA repair; in malignancy, persistent stress induces overexpression, enhancing repair mechanisms but exacerbating genomic instability [69]. Studies report conflicting roles: Overexpression correlates with proliferation, chemoresistance, and reduced survival [70], whereas inhibitors suppress proliferation/migration [71, 72]. Other data show decreased expression [73, 74] or autophagy‐induced cell death upon activation [75, 76]. In neural stem cells, SIRT1 promotes tumorigenesis by inhibiting p53‐dependent surveillance [77]. These contradictions necessitate investigating context‐specific regulatory mechanisms to advance targeted therapies.

As the prototypical epigenetic regulator of genomic stability, aging, apoptosis, and senescence in normal cells—and a dual‐function modulator in malignancy—SIRT1 demonstrates neuroprotective effects in CNS injury. Given its established pathological significance in brain tumors, SIRT1 critically contributes to MGM pathogenesis.

Notably, the integration of our research findings with existing literature indicates that SIRT1 represents a highly promising therapeutic target for MGM; however, its clinical translation still faces several potential challenges. One primary concern involves the pharmacokinetic properties of SIRT1‐modulating compounds, specifically their ability to cross the blood–brain barrier (BBB) to reach intracranial tumors. Numerous SIRT1 modulators have been reported. For instance, SRT1720, a well‐characterized SIRT1 activator, has demonstrated efficacy in a paraquat (PQ)‐induced Parkinson's disease mouse model. Intraperitoneal administration of SRT1720 significantly attenuated PQ‐induced toxic damage to dopaminergic neurons in the substantia nigra and improved motor coordination deficits associated with PQ exposure [78]. Among agents that downregulate SIRT1 protein expression or activity, EX‐527, a potent and selective SIRT1 inhibitor, delivered via intraperitoneal injection, has been shown to mitigate perturbations in the activity of necroptosis‐associated metabolic enzymes downstream of ischemic injury, reduce cerebral infarct volume, and improve survival rates in models of cerebral ischemia [79]. However, despite evidence of effects from various SRT1 modulators in preclinical models of neurological disorders, systematic data on their bioavailability and distribution specifically within the context of MGM remain scarce. Consequently, the development of novel SIRT1‐targeting agents with optimized BBB penetration profiles holds significant potential for future applications.

A second critical consideration pertains to potential off‐target effects and safety concerns. Given that SIRT1 participates in multiple signaling pathways, its systemic activation or inhibition carries a risk of unintended consequences. Nevertheless, current evidence from in vivo studies often reports limited adverse effects. Resveratrol, a natural activator of SIRT1, has been confirmed by numerous studies to exert multiple neuroprotective effects in Alzheimer's disease models through targeting SIRT1, including antioxidant, anti‐inflammatory, and mitochondrial protective actions, as well as interference with Aβ formation [80, 81]. Concurrently, research indicates that resveratrol can help maintain BBB integrity by reducing matrix metalloproteinase‐9 (MMP‐9) and inducing adaptive immune responses, potentially promoting recovery from Aβ deposition in the brain [82].

Future research efforts should prioritize rigorous preclinical validation of SIRT1‐targeting strategies in advanced MGM models, with a focus on systematic pharmacokinetic evaluation, assessment of efficacy, and safety profiling in higher‐order animal models. Addressing these aspects is crucial for bridging the gap between our mechanistic understanding of SIRT1's role in MGM and its potential clinical application.

## 5 Limitation

This study is constrained by several limitations. First, our conclusions predominantly rely on computational data analyses and modeling, corroborated only by a limited number of molecular‐level experiments. While these approaches yield valuable insights, they fail to comprehensively elucidate the intricate biological mechanisms underpinning MGM pathogenesis. Substantiating and validating these findings necessitates additional in vitro and in vivo experimental investigations.

Second, the transcriptomic datasets utilized in this investigation exhibited relatively restricted sample sizes, potentially compromising the robustness, generalizability, and external validity of our predictive models. Consequently, extrapolation of these findings to broader clinical contexts warrants cautious interpretation. We anticipate that future studies incorporating larger and more diverse datasets will enhance model optimization and clinical applicability.

Third, although our correlation‐based analysis identified SIRT1 as a pivotal aging‐associated gene potentially implicated in MGM development, the precise molecular mechanisms linking SIRT1 to tumorigenesis remain incompletely characterized. Moreover, the causal directionality between aging and MGM pathogenesis remains undetermined—specifically, whether aging potentiates tumorigenesis or tumor‐associated molecular alterations accelerate aging phenotypes. Disentangling this bidirectional relationship demands more comprehensive mechanistic exploration.

Collectively, while this study provides novel insights into the aging‐MGM association—particularly SIRT1's potential role—it underscores the imperative for further experimental validation and longitudinal research to establish causal relationships and delineate the underlying biological pathways.

## 6 Prospects for Future Work

Based on the significant differential expression of the aging‐related gene SIRT1 observed in our study and its strong correlation with MGM pathogenesis, we plan to conduct further functional experiments to elucidate the precise role of SIRT1 in MGM development. Our current findings have clearly established associations between SIRT1 expression levels and MGM progression as well as tumor immune infiltration. Building on these results, we intend to perform systematic in vitro and in vivo functional assays, including SIRT1 knockdown and overexpression models, to evaluate the impact of this gene on core cellular processes in MGM, such as proliferation, apoptosis, invasion, and immunomodulation within the tumor microenvironment.

This experimental direction represents a natural extension of our current work and is crucial for advancing from observational correlations to mechanistic insights. Elucidating the functional role of SIRT1 will significantly contribute to the overarching objective of this project—identifying and validating aging‐related biomarkers with prognostic and therapeutic relevance in MGM. We are actively preparing for subsequent investigations and are confident that these studies will substantially strengthen the biological plausibility of SIRT1 as a biomarker, facilitate its clinical translation, and potentially provide new targets for therapeutic intervention.

## 7 Conclusion

In conclusion, this study conducted a systematic assessment of the association between ARGs and MGM through an integrated methodological framework encompassing bioinformatics analysis, machine learning algorithms, MR, and experimental validation. We identified SIRT1 as a pivotal aging‐associated gene demonstrating significant association with MGM pathogenesis. Moreover, these findings unveil novel therapeutic avenues for future interventions and personalized medicine by highlighting potential molecular targets within aging‐related pathways.

## Ethics Statement

This study and the acquisition of human tissue specimens were approved by the Medical Ethics Committee of the Affiliated Hospital of Qingdao University (Approval No. QYFYwzll30035), and were conducted in full compliance with the Declaration of Helsinki.

## Consent

The authors have nothing to report.

## Conflicts of Interest

The authors declare no conflicts of interest.

## Supporting information


**Figure S1:** Full uncropped Western blot images for three independent biological replicates corresponding to Figure 8. (A) Cropped and incorporated into the main manuscript body.
**Table S1:** Primer sequences for SIRT1 and GAPDH.

## Data Availability

The original contributions presented in the study are included in the article. Further inquiries can be directed to the corresponding author.
