# Supplementary material for: Integrated Multiomics Analysis and Mendelian Randomization Identify SIRT1 as a Pivotal Aging‐Associated Gene in Meningioma
Source: IUBMB Life. 2025 Nov 10;77(11):e70072. doi: 10.1002/iub.70072 (PMC12598521; doi:10.1002/iub.70072)
Supplement: Supplementary file 1 — Figure S1: Full uncropped Western blot images for three independent biological replicates corresponding to Figure 8. (A) Cropped and incorporated into the main manuscript body. Table S1: Primer sequences for SIRT1 and GAPDH. [file IUB-77-0-s001.docx]

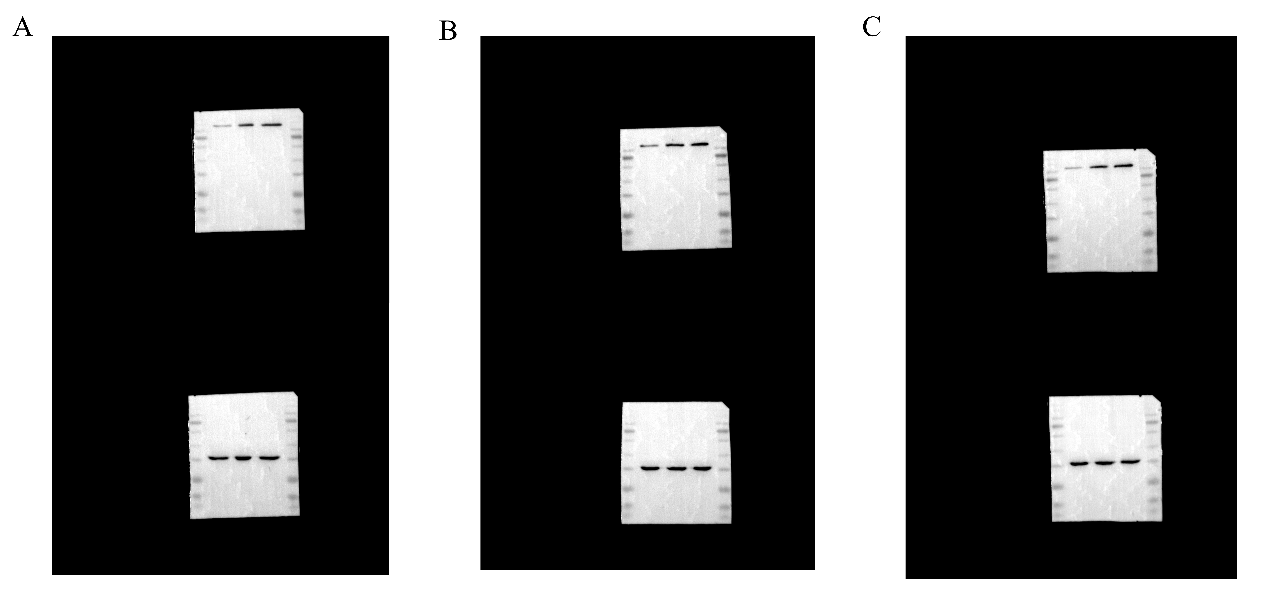


Supplementary Figure S1. Full uncropped Western blot images for three independent biological replicates corresponding to Fig. 8. Panel A was cropped and incorporated into the main manuscript body.

Supplementary Table S1. Primer sequences for SIRT1 and GAPDH.

| Primer Name | Primer Sequence |
| --- | --- |
| H-SIRT1-F | TAGCCTTGTCAGATAAGGAAGGA |
| H-SIRT1-R | ACAGCTTCACAGTCAACTTTGT |
| H-GAPDH-F | GACCTGACCTGCCGTCTA |
| H-GAPDH-R | AGGAGTGGGTGTCGCTGT |
